# Severe Idiopathic Secretory Diarrhoea With a Profound Sustained Response to Somatostatin Analogues: A Case Report

**DOI:** 10.1155/crgm/7006260

**Published:** 2025-10-28

**Authors:** Geovanny Gandy, Alexander James Anthony Prudence, Miriam Tania Levy

**Affiliations:** ^1^Gastroenterology Department, Liverpool Hospital, Liverpool, New South Wales, Australia; ^2^University of NSW, Sydney, New South Wales, Australia; ^3^Gastroenterology Department, Coffs Harbour Health Campus, Coffs Harbour, New South Wales, Australia

**Keywords:** chronic secretory diarrhoea, lanreotide, octreotide, VIPoma

## Abstract

**Background:**

Chronic secretory diarrhoea is a diagnostic challenge with a broad differential and significant impact on patient's quality of life. While common causes include microscopic colitis, bile acid diarrhoea, and laxative use, rarer aetiologies such as vasoactive intestinal peptide (VIP)–secreting neuroendocrine tumours (VIPomas) must be considered when standard investigations fail.

**Case Presentation:**

We present a 35-year-old woman with a two-year history of progressively worsening, fasting-persistent, high-volume watery diarrhoea leading to severe electrolyte abnormalities and weight loss requiring resuscitation in intensive care. Extensive biochemical, endoscopic, and radiological investigations—including faecal analysis, colonoscopy, neuroendocrine markers, multiphase CT, endoscopic ultrasound, and Ga-68 Dotatate PET imaging—failed to identify an underlying cause. Serum VIP levels remained within the normal range. Despite the absence of a definitive diagnosis, empirical treatment with the somatostatin analogue octreotide led to rapid and sustained symptom resolution. The patient was subsequently maintained on long-acting lanreotide with complete remission. Notably, diarrhoea recurred upon cessation of therapy, again resolving with reinitiation. After 4 years, the patient self-ceased lanreotide without symptom recurrence, and follow-up imaging remained unremarkable.

**Discussion:**

This case highlights a diagnostic dilemma: clinical and biochemical features were highly suggestive of a VIPoma, yet no tumour was identified despite repeated advanced imaging and biochemical workup. The patient's remarkable therapeutic response to somatostatin analogue therapy, in the absence of confirmed neuroendocrine neoplasia, suggests that somatostatin analogues may have a broader role in the management of idiopathic secretory diarrhoea than currently appreciated.

**Conclusion:**

We present a rare case of chronic secretory diarrhoea with suspected but unproven VIPoma, demonstrating sustained and reproducible response to somatostatin analogue therapy. This case supports the consideration of therapeutic trials of somatostatin analogues in refractory secretory diarrhoea of unknown origin.

## 1. Introduction

Chronic secretory diarrhoea has a wide range of differentials and can cause significant impact on patients' quality of life. Patient outcomes are usually dependent on the identification and treatment of the causative aetiology via biochemical, endoscopic and microbiological testing. Its most common causes include laxative abuse, bile salt diarrhoea and microscopic colitis with rarer causes including hormone-secreting neuroendocrine tumours.

Vasoactive intestinal peptide (VIP)–secreting tumours are a rare cause of secretory diarrhoea with an incidence of 1 in 10 million people per year [1]. While the vast majority of VIPomas are located within the pancreas, other VIP-secreting extrapancreatic neoplasms have been reported, most commonly arising from the sympathetic chain [1]. These neuroendocrine neoplasms autonomously secrete VIP which acts on intestinal epithelial cells to induce fluid and electrolyte secretion into the lumen. This manifests in a distinct clinical triad of watery diarrhoea, hypokalaemia and achlorhydria [1]. Diarrhoea is profuse and secretory in nature with volumes up to 3 L per day and a faecal osmolar gap of less than 50.

Diagnosis of this rare condition can be challenging. While an elevated serum VIP level greater than 20 pmol/L is highly specific, VIPomas may only secrete VIP intermittently and levels may not be elevated between episodes of diarrhoea due to the peptide's short half-life [2]. Recommended imaging modalities for localising and staging these tumours include computed tomography (CT), magnetic resonance imaging (MRI), endoscopic ultrasound (EUS), as well as ^68^Ga/^64^Cu-DOTA-somatostatin analogue positron emission tomography (Ga-68 DOTATE PET) which has the highest sensitivity of 97% [2].

We present a case of chronic secretory diarrhoea with clinical features compatible with a diagnosis of a VIPoma. Despite negative investigations, and failure to identify a causative aetiology, the patient had a rapid and sustained response to somatostatin analogue therapy. This case enriches the literature by illustrating the diagnostic pitfalls in evaluating chronic diarrhoea when standard investigations return normal results. It emphasises the importance of maintaining clinical suspicion for occult neuroendocrine processes, even in the absence of biochemical or radiological confirmation.

## 2. Case Presentation

**Figure 1:**
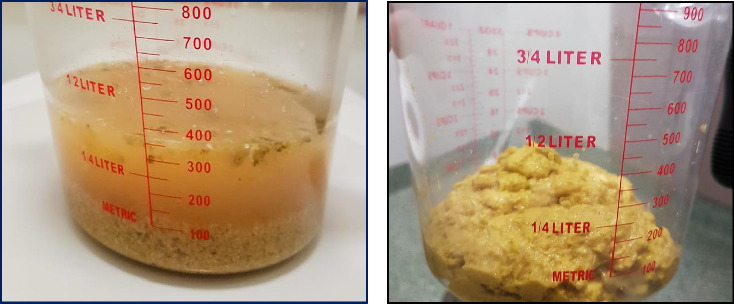
Patient stool consistency before and after treatment with octreotide. (a) Patient stool sample during admission. Prior to commencement of octreotide, (b) stool sample after commencement of octreotide.

A 35-year-old Vietnamese female presented with progressively worsening profuse nonbloody diarrhoea up to 10 times daily with crampy abdominal pain and 10 kg of weight loss (Figure 1(a)). This was on a background of 2 years of at least four loose watery bowel motions a day. She reported no fevers, infective symptoms, sick contacts, recent antibiotic use, or recent overseas travel. The diarrhoea had no relationship to food intake and persisted independent of fasting. Her past medical history includes immune thrombocytopenia previously managed with azathioprine 50 mg daily and romiplostim 350 mcg weekly prior to a splenectomy 8 years prior to the diarrhoea occurring. The patient also reported gastro-oesophageal reflux disease, and cholecystectomy the year prior, 1 year after the commencement of the diarrhoeal illness. She had no relevant family history. The patient denied use of laxatives and had no regular medications associated with inducing diarrhoea.

On presentation, the patient was hypotensive with a blood pressure of 90/60 with severe electrolyte derangements including hypokalaemia 1.7 mmol/L with associated U-waves and prolonged QT on ECG. This was accompanied by hypomagnesaemia of 0.62 mmol/L, hypophosphatasemia of 0.7 mmol/L, a high anion gap metabolic acidosis of 22 mmol/L and a mild acute kidney injury with creatinine of 85 μmol/L (baseline 50 μmol/L). She was admitted to the intensive care for resuscitation and management.

Her diarrhoea was extensively investigated (Table 1). Stool microscopy, extended PCR and cultures were negative, faecal calprotectin was not raised, and faecal elastase was normal. Stools were nonbloody, watery and profuse exceeding 2.5L per day of stool output despite fasting. Faecal chemistry showed a sodium of 72 mmol/L (reference 30 mmol/L) with a calculated osmolar gap of 16 mOsm/kg (normal reference 50–75 mOsm/kg) consistent with secretory diarrhoea. Gastroscopy and colonoscopy were unremarkable macroscopically. Gastric, small bowel and colonic biopsies showed mild focal subtotal villous blunting without increase in intraepithelial lymphocytes, and normal tubular architecture—excluding coeliac disease, microscopic colitis, and inflammatory bowel disease. Thyroid function was within normal range, and tuberculosis gamma interferon assay and coeliac disease serology were both negative. Human immunodeficiency virus serology was negative too. A neuroendocrine screen, comprising 24 h urine 5-HIAA, VIP, chromogranin A, gastrin, and plasma and urine metanephrines and normetanephrines, was normal. Serum VIP level was 16.9 pmol/L which is within the normal range < 50 pmol/L. Stool bile acids were normal. A multiphase CT scan of her abdomen and pelvis, EUS and Ga-68 Dotatate PET scan failed to identify any neuroendocrine lesions or malignant pathology.

The patient was initially commenced on cholestyramine and loperamide with mild improvement in frequency of bowel motions but persistent hypokalemia and hypochloraemia. Subcutaneous octreotide 50 μg twice a day was trialled with remarkable improvement in stool frequency, consistency and resolution of electrolyte abnormalities (Figure 1(b)). The patient was transitioned to long-acting depot lanreotide 120 mg, to improve compliance and long-term maintenance and discharged home with three formed stools per day and stable electrolytes. This was well tolerated generally aside from mild dryness of mucous membranes which was treated with artificial saliva. Two years later, on self-cessation of lanreotide, the profuse watery diarrhoea and electrolyte abnormalities recurred, resulting in readmission. Repeat investigations for VIPoma at this time produced VIP levels within the normal range and a second negative Ga-68 DOTATE PET scan. This response was recovered with recommencement of the somatostatin analogue, allowing her to regain her independence and return to usual work. After 4 years, the patient ceased lanreotide without recurrence. A formal diagnosis was never made.

## 3. Discussion

Chronic diarrhoea is a common gastrointestinal complaint, classically defined as the passage of loose stools (between Type 5 and 7 on the Bristol stool chart) at an increased frequency for a duration of more than 4 weeks. Characterising the type of diarrhoea (see Table 2) based on features identified on history and initial tests can guide further investigations and narrow down differential causes. Secretory diarrhoea is typically watery, nonbloody and large volume stool that persists during fasting. Its most common causes include laxative abuse, bile salt diarrhoea and microscopic colitis. Rarer causes include hormone-secreting neuroendocrine tumours such as carcinoid syndrome, gastrinoma and VIPoma.

In this case, clinical features and stool chemistry were suggestive of a secretory diarrhoea with a low faecal osmolar gap. Negative colonoscopy ruled out adenoma or microscopic colitis as the culprit. While azathioprine-induced villous architectural distortion resulting in chronic diarrhoea has been described in isolated reports [4], it usually resolves with cessation and so is unlikely in this case. Our patient's symptoms preceded her cholecystectomy and despite mild symptomatic improvement on commencement of both cholestyramine and loperamide, postcholecystectomy diarrhoea seemed unlikely explanation based on timing and the severity of symptoms. Despite the lack of radiological confirmation, the temporal relationship between our patient's somatostatin blockade and rapid resolution of diarrhoea and severity of electrolyte abnormalities was initially concerning for a VIPoma.

Diagnosis of VIPoma is usually delayed, and most pancreatic VIPomas are greater than 3 cm at presentation with 60%–80% already metastasised [5]. While multiphase CT is highly accurate (sensitivity > 80%) in detection of primary pancreatic neuroendocrine tumours, functional PET imaging with Ga-68 DOTATATE or Ga-68 DOTATOC has increased sensitivity in detection of small lesions [5]. EUS can also detect smaller pancreatic tumours and allow for transmucosal needle biopsy but does not allow evaluation of extra-pancreatic lesions. In our case, these imaging modalities did not find a VIPoma. Multiphase CT of pancreas and Ga-68 DOTATATE were repeated 2 years later after a relapse in diarrhoea on cessation of lanreotide, and these were similarly unremarkable. Despite the complexities in diagnosis, it seems unlikely that an occult VIPoma was the cause given the extended period of clinical and radiological follow-up.

Some suggest a trial of somatostatin analogue octreotide for the treatment of secretory diarrhoea of unknown aetiology where all standard antidiarrhoeal agents have failed. Despite evidence for its use in postchemotherapy refractory diarrhoea, and some potentially promising results in small studies for diarrhoea related to postgastrectomy dumping syndrome and short gut syndrome, this hypothesis is not yet supported [6]. To the best of our knowledge, no recent studies report significant benefit in the treatment of refractory diarrhoea with octreotide outside these aetiologies.

The aetiology of this patient's chronic profuse secretory diarrhoea could not be concluded biochemically, endoscopically or radiologically. The strength of this case lies in its detailed characterisation of a patient with chronic secretory diarrhoea who responded dramatically to somatostatin analogue therapy. This reproducible therapeutic response, including relapse upon cessation, reinforces the value of somatostatin analogues in otherwise unexplained cases. However, a key limitation is the lack of histopathological confirmation of a VIPoma or other neuroendocrine tumour, which limits diagnostic certainty and generalisability. Additionally, spontaneous symptom resolution after 4 years off therapy raises the possibility of a transient or functional neuroendocrine process not captured by current diagnostic modalities.

This case challenges the notion that treatment must be diagnosis-dependent in all cases of secretory diarrhoea. It illustrates that somatostatin analogues remain a viable treatment consideration for secretory diarrhoea even when the aetiology remains elusive. While empirical therapy should not replace thorough diagnostic workup, it may provide relief in select cases of idiopathic secretory diarrhoea when quality of life is severely impacted.

## Figures and Tables

**Table 1:** Summary of investigations for chronic secretory diarrhoea.

Category	Investigation	Result	Reference range	Interpretation/remarks
Stool investigations	Microscopy, culture and extended PCR	Negative	—	Infectious causes excluded
	Calprotectin	Not raised	< 50 μg/g	Inflammatory cause unlikely
	Elastase	Normal	> 200 μg/g	Pancreatic insufficiency excluded
	Stool volume	> 2.5 L/day despite fasting	> 1 L/day (secretory)	Suggestive of secretory diarrhoea
	Sodium	72 mmol/L	> 30 mmol/L (secretory)	Consistent with secretory diarrhoea
	Osmolar gap	16 mOsm/kg	< 50 mOsm/kg (secretory)	Supports secretory mechanism
	Bile acids	Normal	—	Bile acid diarrhoea unlikely

Serum/biochemical	Potassium	1.7 mmol/L	3.5–5.2 mmol/L	Severe hypokalaemia
	Magnesium	0.62 mmol/L	0.7–1.1 mmol/L	Hypomagnesaemia
	Phosphate	0.7 mmol/L	0.75–1.5 mmol/L	Hypophosphataemia
	Anion gap	22 mmol/L	8–16 mmol/L	High anion gap metabolic acidosis
	Creatinine	85 μmol/L (baseline 50)	45–90 μmol/L	Mild AKI
	Thyroid function tests	Normal	—	Thyrotoxicosis excluded
	Coeliac serology	Negative	—	Coeliac disease unlikely
	TB gamma interferon assay	Negative	—	Excludes latent tuberculosis
	HIV serology	Negative	—	HIV-related diarrhoea unlikely
	Serum VIP	16.9 pmol/L	< 50 pmol/L	Within normal limits
	24 h urine 5-HIAA	Normal	< 45 μmol/day	Carcinoid syndrome unlikely
	Chromogranin A	Normal	< 100 ng/mL	No biochemical evidence of NET
	Gastrin	Normal	< 100 pmol/L	Gastrinoma unlikely
	Plasma and urine metanephrines/normetanephrines	Normal	—	Rule out pheochromocytoma/paraganglioma

Endoscopic	Gastroscopy and colonoscopy	Macroscopically unremarkable	—	No visible pathology
	Histology (gastric, small bowel, colon)	Mild focal villous blunting, no increase in IELs, normal architecture	—	Coeliac, microscopic colitis, IBD excluded

Imaging/functional	Multiphase CT abdomen/pelvis	No lesions identified	—	No structural NET seen
	Endoscopic ultrasound	No masses or pancreatic lesions	—	Small pancreatic NET excluded
	Ga-68 DOTATATE PET	No uptake suggestive of neuroendocrine tumour	—	High sensitivity test negative; NET unlikely

**Table 2:** Classification and characteristic of chronic diarrhoea subtypes [3].

Classification	Stool osmolar gap^∗^	Stool characteristics	Common aetiologies	Additional tests
Secretory	< 50 mOsm/kg	Large volume watery stool (> 3 L/24 h) persists during fasting	Microscopic colitis Bile acid malabsorption Neuroendocrine tumours Nonosmotic laxatives (e.g., senna, docusate) Bacterial enterotoxins (e.g., cholera)	Endoscopy and histology Neuroendocrine screen Stool bile acids

Osmotic	> 75 mOsm/kg	Less voluminous than secretory and improves with fasting	Osmotic laxatives Carbohydrate malabsorption	Stool Na concentration < 70 mEq/L

Inflammatory	Variable	Liquid loose stools with erythrocytes and leukocytes	Invasive bacterial or parasitic infections Pseudomembranous colitis Colonic inflammatory bowel disease Ischaemic colitis Radiation colitis	Stool culture and C. difficile toxin Increased faecal calprotectin Increased serum CRP and WCC

Malabsorption	Variable	Often steatorrhea	Exocrine pancreatic insufficiency Coeliac disease Small intestinal bacterial overgrowth Fistulising and ileal Crohn's disease Short bowel syndrome	Increased faecal elastase Faecal fat stain positive Biochemical evidence of nutritional deficiencies

^∗^Osmolar gap = faecal osmolality − 2 × (faecal sodium + faecal potassium).

## Data Availability

Data sharing is not applicable to this article as no datasets were generated or analysed during the current study.
